# Sex differences among patients with transthyretin amyloid cardiomyopathy – from diagnosis to prognosis

**DOI:** 10.1002/ejhf.2646

**Published:** 2022-08-16

**Authors:** Rishi K. Patel, Adam Ioannou, Yousuf Razvi, Liza Chacko, Lucia Venneri, Francesco Bandera, Daniel Knight, Tushar Kotecha, Ana Martinez‐Naharro, Ambra Masi, Aldostefano Porcari, James Brown, Kiara Patel, Charlotte Manisty, James Moon, Dorota Rowczenio, Janet A. Gilbertson, Gianfranco Sinagra, Helen Lachmann, Ashutosh Wechalekar, Aviva Petrie, Carol Whelan, Philip N. Hawkins, Julian D. Gillmore, Marianna Fontana

**Affiliations:** ^1^ National Amyloidosis Centre, Division of Medicine University College London London UK; ^2^ Heart Failure Unit, Cardiology University Department IRCCS Policlinico San Donato Milan Italy; ^3^ Department for Biomedical Sciences for Health University of Milano Milan Italy; ^4^ Center for Diagnosis and Treatment of Cardiomyopathies, Cardiovascular Department Azienda Sanitaria Universitaria Giuliano‐Isontina (ASUGI), University of Trieste Trieste Italy; ^5^ Barts Heart Centre, The Cardiovascular Magnetic Resonance Imaging Unit, and the Inherited Cardiovascular Diseases Unit St Bartholomew's Hospital London UK; ^6^ Eastman Dental Institute, University College London London UK

**Keywords:** Amyloidosis, Echocardiography, Sex, Diagnosis, Prognosis

## Abstract

**Aims:**

Transthyretin amyloid cardiomyopathy (ATTR‐CM) is predominantly diagnosed in men. The few available studies suggest affected women have a more favourable cardiac phenotype. We aimed to characterize sex differences among consecutive patients with non‐hereditary and two prevalent forms of hereditary (h)ATTR‐CM diagnosed over a 20‐year period.

**Methods and results:**

Analysis of deep phenotyping at presentation, changes on serial echocardiography and overall prognosis were evaluated. In total, 1732 consecutive patients were studied, comprising: 1095 with wild‐type (wt)ATTR‐CM; 206 with T60A‐hATTR‐CM; and 431 with V122I‐hATTR‐CM. Female prevalence was greater in T60A‐hATTR‐CM (29.6%) and V122I‐hATTR‐CM (27.8%) compared to wtATTR‐CM (6%). At presentation, females were 3.3 years older than males (wtATTR‐CM: 81.9 vs. 77.8 years; T60A‐hATTR‐CM: 68.7 vs. 65.1 years; V122I‐hATTR‐CM: 77.1 vs. 74.9 years). Body size significantly influenced measures of disease severity; when indexed, overall structural and functional phenotype was similar between sexes, the few significant differences suggested a mildly worse phenotype in females. No significant differences were observed in both disease progression on serial echocardiography and mortality across the overall population (*p* = 0.459) and when divided by genotype (wtATTR‐CM: *p* = 0.730; T60A‐hATTR‐CM: *p* = 0.161; V122I‐hATTR‐CM: *p* = 0.056).

**Conclusion:**

This study of a well‐characterized large cohort of ATTR‐CM patients did not demonstrate overall differences between sexes in either clinical phenotype, when indexed, or with respect to disease progression and prognosis. Non‐indexed wall thickness measurements may have contributed to both under‐representation and delays in diagnosis for affected females and highlights the potential role of utilizing indexed echocardiographic parameters for a more accurate assessment of patients at diagnosis and for disease prognostication.

## Graphical abstract

Deep echocardiographic phenotyping of 1732 patients with ATTR‐CM found no differences in the clinical phenotype between males and females when taking body size into account, with no differences in disease progression on serial imaging and no difference in mortality. Current clinical practice adopts the use of non‐indexed wall thickness measurements, which may have contributed to females being under‐represented, presenting later than males and often with a mildly worse phenotype.

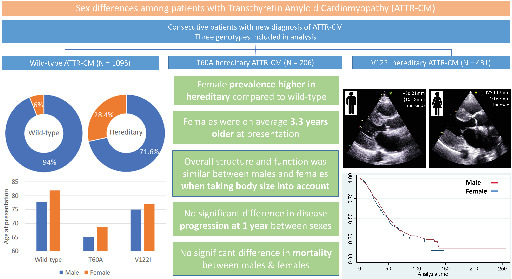

## Introduction

Transthyretin amyloid cardiomyopathy (ATTR‐CM) is a progressive, life‐threatening disease caused by deposition of misfolded aggregated transthyretin (TTR) in the form of amyloid fibres, which accumulate within the myocardial extracellular space and give rise clinically to heart failure.^1^, ^2^ ATTR‐CM may be sporadic or familial.^3^ The non‐inherited, wild‐type form (wtATTR‐CM) is the most common, presents later in life and has a median survival of 3–5 years. The hereditary form (hATTR‐CM) is associated with TTR gene mutations and often presents earlier in life; it has an autosomal dominant pattern of inheritance with variable penetrance, whilst the course of the disease and survival vary within families and between different genotypes.

Male predominance in ATTR‐CM is well recognized. Studies generally report more than 85% of wtATTR‐CM patients are male, as are the majority of patients with hATTR‐CM including those with T60A and V122I,^4^, ^5^, ^6^, ^7^, ^8^ which prevail in the UK. However, the literature is unclear regarding differences in disease severity and survival among affected men and women. The largest study to date reported a more severe phenotype in men based on a limited number of echocardiographic parameters.^9^ However, the majority of patients included in this non‐interventional, observational study only had the V30M genetic variant. Furthermore, the presence of cardiac involvement was defined based on a single measure of left ventricular (LV) wall thickness of >12 mm on echocardiogram. Although this leaves uncertainty about other causes of increased LV mass (LVM), the diagnosis of ATTR‐CM was determined by an expert physician who assessed the diagnosis based on multiple clinical parameters such that the likelihood of cardiac involvement for this cohort was high. Retrospective studies of scintigraphy‐confirmed ATTR‐CM similarly report a favourable phenotype among women with both wild‐type and hereditary subtypes but were restricted by both small sample sizes and the limited echocardiographic parameters studied.^7^, ^10^, ^11^, ^12^ In contrast, a recent study actually demonstrated a worse echocardiographic phenotype in women compared to men for both wild‐type and hereditary subtypes.^13^ This study also determined no differences in all‐cause mortality between sexes, which was also noted in another small single‐centre study in V122I patients.^7^ The prognostic data of these two studies were limited by a follow‐up period of 12 to 18 months. Overall, considering the limitation of the current literature,^14^, ^15^, ^16^, ^17^, ^18^, ^19^ differences in phenotypes, disease progression and long‐term prognosis remain uncertain.

The study we report here comprises the largest single cohort of patients with ATTR‐CM to date and aims to compare male and female patients with respect to: (i) phenotypic differences and baseline echocardiographic parameters across the common genotypes of ATTR‐CM, (ii) disease progression through changes in structural and functional parameters on serial echocardiography studies over time; and (iii) differences in overall prognosis.

## Methods

Consecutive patients with a diagnosis of ATTR‐CM referred to the National Amyloidosis Centre (NAC), Royal Free Hospital, London, UK between 1 January 2000 and 22 June 2021 were included. Between 2000 and 2005, the diagnosis of ATTR‐CM was determined based on heart failure symptoms alongside a characteristic echocardiogram, and either direct endomyocardial biopsy proof of ATTR amyloid or ATTR amyloid in an extra‐cardiac biopsy. From 2006, cardiovascular magnetic resonance was added to the assessment in cases of diagnostic uncertainty following echocardiography. From 2010 onwards, 99mTechnetium labelled 3,3‐diphosphono‐1,2‐propanodicarboxylic acid (99mTc‐DPD) scintigraphy was utilized, and a diagnosis established based on ATTR amyloid in an extra‐cardiac biopsy with cardiac uptake on 99mTc‐DPD scintigraphy; or grade 2–3 cardiac uptake on 99mTc‐DPD scintigraphy in the absence of biochemical evidence of a plasma cell dyscrasia.^20^ All patients underwent sequencing of the transthyretin (*TTR*) gene. The study analysis was limited to the three most common genotypes in the UK: wtATTR‐CM, T60A‐hATTR‐CM and V122I‐hATTR‐CM. Other hereditary genotypes were not included in this analysis due to limited patient numbers. NAC staging is a validated staging system used to estimate prognosis for patients with ATTR‐CM at the time of diagnosis.^21^ This was determined for each patient based on N‐terminal pro‐B‐type natriuretic peptide (NT‐proBNP) level and estimated glomerular filtration rate (eGFR).^21^ Patients were managed in accordance with the Declaration of Helsinki and provided written informed consent for analysis and publication of their data (REC reference 21/PR/0620).

### Echocardiography

All echocardiograms were performed using a GE ultrasound machine with a 5S probe and analysed offline using EchoPAC software (the most up‐to‐date version used at the time of acquisition) by qualified operators in accordance with current guidelines^22^ and blinded to both sex and disease genotype. Valvular regurgitation was deemed significant if at least moderate. The thickness of the LV septum and posterior wall was measured using two‐dimensional parasternal long‐axis views to avoid angle‐dependent misalignment.

### Statistical analysis

IBM SPSS Statistics Version 25 (IBM) was used for all statistical analyses apart from the survival analyses when Stata (StataCorp. 2021. Stata Statistical Software: Release 17: StataCorp LLC, College Station, TX, USA) was used. All numerical variables were tested for normality (Shapiro–Wilk test) and are presented as mean (standard deviation [SD]) or median (interquartile range), other than NT‐proBNP which was natural log (ln) transformed for bivariate testing. The independent *t‐*test or its non‐parametric equivalent (Mann–Whitney U test) were used to compare means or distributions, respectively, of two groups, and the one‐way analysis of variance (ANOVA) or its non‐parametric equivalent (Kruskal–Wallis test) were used to compare means or distributions, respectively, of multiple groups, with a significant result followed by a post‐hoc Bonferroni corrected pairwise comparison to establish where differences lay. Levene's test was used to check the homogeneity of variance in *t*‐test and ANOVA. Categorical data are presented as absolute numbers and frequencies and compared using the Chi‐squared test or Fisher exact test, as appropriate. Multivariable analysis of possible determinants of wall thickness by linear regression was also conducted to assess the independent contribution of sex. Linear regression analysis was used for all the numerical variables at 12 months to allow a comparison of the genotypes after adjusting for age at baseline and the baseline value of the variable. The assumptions of the regression analyses were checked by a study of the residuals. Mortality data were obtained via the UK Office of National Statistics (ONS). For English patients, outcome data were obtained from the ONS on a monthly basis but may be longer for non‐English UK patients. The mortality endpoint was defined as time to death from baseline for all deceased patients and time to censor date, 22 October 2021, from baseline among the remainder. Non‐UK patients were censored at last contact unless known to be deceased. Survival was evaluated with Cox proportional hazards regression analysis, providing estimated hazard ratios (HRs) with 95% confidence intervals (CIs), and Kaplan–Meier curves were drawn. The proportional hazards assumption was checked for each Cox analysis. Statistical significance was defined as a *p*‐value <0.05.

## Results

### Baseline characteristics of all patients at baseline and comparisons between different genotypes

The study cohort comprised 1732 patients. The majority had wtATTR‐CM (*n* = 1095, 63.4%, mean age 78.1 years), 206 patients (11.9%) had T60A‐hATTR‐CM (mean age 66.2 years) and 431 patients (24.9%) had V122I‐hATTR‐CM (mean age 75.5 years).

**Figure 1:**
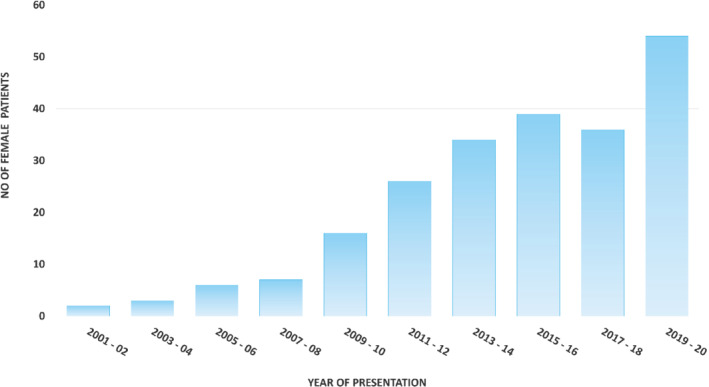
Number of female patients assessed at the National Amyloidosis Centre with a new diagnosis of transthyretin amyloid cardiomyopathy. Patients divided into groups of 2‐year blocks. Patient data from 1 January 2021 to censor date were not included in this graph as this represents only an 8‐month period of time.

Overall, 1485 patients (85.9%) were male. When split by genotype, 1029 (94%) wtATTR‐CM patients, 145 (70.4%) T60A‐hATTR‐CM patients and 311 (72.2%) V122I‐hATTR‐CM patients were male (*Table* *1*). Across all genotypes, female patients were significantly older at presentation (81.9 vs. 77.8 years in wtATTR‐CM; 68.7 vs. 65.1 years in T60A‐hATTR‐CM; 77.1 vs. 74.9 years in V122I‐hATTR‐CM) and had a lower body surface area (BSA) than males (mean BSA 1.65 m^2^ in females vs. 1.97 m^2^ in males). Serum NT‐proBNP concentration was similar between sexes (median NT‐proBNP 2791 ng/L [1497–5193 ng/L] in males vs. 2799 ng/L [1501–5190 ng/L] in females across all genotypes). Estimated GFR was significantly higher in men compared to women with wtATTR‐CM (60 vs. 46 ml/min, *p* < 0.001). Mean troponin was significantly higher in men compared to women with V122I‐ATTR‐CM (83 vs. 58 ng/L, *p* < 0.001). In terms of medical therapy for heart failure, there was no significant difference between sexes and across all genotypes (*Table* *1*). *Figure* *1* demonstrates the substantial increase in diagnosis of female patients with ATTR‐CM since 2001.

**Table 1:** Baseline characteristics of patients with wild‐type, T60A and V122I cardiac amyloidosis classified by sex

Variables	Wild‐type (*n* = 1095)			T60A (*n* = 206)			V122I (*n* = 431)		
	Male	Female	*p*‐value	Male	Female	*p*‐value	Male	Female	*p*‐value
Prevalence	1029 (94.0%)	66 (6.0%)	<0.001	145 (70.4%)	61 (29.6%)	<0.001	311 (72.2%)	120 (27.8%)	<0.001
Age (years)	77.80 (6.62)	81.85 (5.8)	<0.001	65.14 (6.51)	68.67 (5.64)	<0.001	74.89 (7.65)	77.08 (5.54)	0.001
BSA (m^2^)	1.94 (0.17)	1.66 (0.15)	<0.001	2.07 (1.71)	1.60 (0.15)	0.002	1.91 (0.18)	1.70 (0.17)	<0.001
Biomarker stage									
Stage 1	474 (46.1%)	19 (28.8%)	0.007	91 (62.8%)	32 (52.5%)	0.170	133 (42.8%)	50 (41.7%)	0.836
Stage 2	375 (36.4%)	19 (28.8%)	0.201	32 (22.1%)	18 (29.5%)	0.259	111 (35.7%)	41 (34.2%)	0.771
Stage 3	140 (13.6%)	20 (30.3%)	<0.001	5 (3.4%)	5 (8.2%)	0.143	62 (19.9%)	26 (21.7%)	0.678
Missing data	40 (3.8%)	8 (12.1%)	0.001	17 (11.7%)	6 (9.8%)	0.693	5 (1.6%)	3 (2.5%)	0.545
Blood biomarkers									
NT‐proBNP (ng/L)	2916 (1643–5103)	3205 (1696–5915)	0.122	1544 (808–3662)	2110 (1008–4634)	0.245	3187 (1624–6066)	2799 (1514–5242)	0.771
Troponin T (ng/L)	60 (42–85)	52 (33–76)	0.109	38 (26–60)	36 (21–46)	0.132	83 (56–117)	58 (40–95)	<0.001
eGFR (ml/min)	60 (48–73)	46 (40–64)	<0.001	80 (65–90)	74 (62–90)	0.266	55 (44–66)	51 (38–67)	0.195
Heart failure medication									
Loop diuretics	821 (79.8%)	55 (83.3%)	0.522	87 (60.0%)	32 (52.5%)	0.317	255 (82.0%)	99 (82.5%)	0.902
ACEi/ARB	510 (49.6%)	28 (42.4%)	0.248	35 (24.1%)	11 (18.0%)	0.337	158 (50.8%)	62 (51.7%)	0.872
BB	489 (47.5%)	33 (50%)	0.718	28 (19.3%)	18 (29.5%)	0.109	162 (52.1%)	68 (56.7%)	0.393
MRA	398 (38.7%)	22 (33.3%)	0.374	38 (26.2%)	16 (26.2%)	0.997	137 (44.1%)	56 (46.7%)	0.625
SGLT2i	7 (0.7%)	0 (0%)	0.501	0 (0%)	0 (0%)	N/A	4 (1.3%)	0 (0%)	0.212
ARNI	10 (1.0%)	0 (0%)	0.420	0 (0%)	1 (1.6%)	0.122	8 (2.6%)	4 (3.3%)	0.667

Data are presented as mean (standard deviation), *n* (%), or median (interquartile range).

ACEi, angiotensin‐converting enzyme inhibitor; ARB, angiotensin receptor blocker; ARNI, angiotensin receptor–neprilysin inhibitor; BB, beta‐blocker; BSA, body surface area; eGFR, estimated glomerular filtration rate; MRA, mineralocorticoid receptor antagonist; NT‐proBNP, N‐terminal pro‐B‐type natriuretic peptide; SGLT2i, sodium–glucose cotransporter 2 inhibitor.

### Differences in echocardiographic findings between males and females at baseline and comparisons between genotypes

In total, 85 out of the 1732 patients (5%) had limited echocardiogram studies that were not evaluable. Across a wide range of echocardiographic parameters, the overall differences pointed to either a similar or mildly worse clinical phenotype in females compared to males, characterized by greater LV wall thickness when indexed for BSA and height, a greater degree of diastolic failure, and a more severe degree of mitral and tricuspid regurgitation. Systolic function parameters were similar between sexes. LVM was greater in males compared to females irrespective of whether indexed or not. All parameters studied were adjusted for age.

At diagnosis, female patients had a significantly thinner mean interventricular septum in diastole (IVSd; 16.15 vs. 17.13 mm, *p* < 0.001), smaller mean LV cavity size (measured both by LV end‐diastolic diameter [LVEDD]; smaller mean LV end‐diastolic [LVEDV] and end‐systolic volumes [LVESV]), stroke volume (SV), LVM and right atrial area when compared to males across the entire cohort (*Table* *2*) and when divided by genotype (online supplementary *Table* *S1*).

**Table 2:** Echocardiographic findings at baseline in patients with transthyretin amyloid cardiomyopathy classified by sex

Variables	All patients (*n* = 1732)		
	Male (*n* = 1485)	Female (*n* = 247)	*p*‐value
IVSd (mm)	17.13 (2.34)	16.15 (2.65)	**<0.001**
PWTd (mm)	16.54 (2.46)	15.92 (2.53)	**0.001**
LVEDD (mm)	43.75 (5.84)	39.90 (5.33)	**<0.001**
LVESD (mm)	33.29 (6.29)	29.86 (5.44)	**<0.001**
LVM (g)	317.11 (84.43)	255.31 (80.03)	**<0.001**
MWT (mm)	16.67 (2.78)	15.69 (3.40)	**<0.001**
RWT	0.77 (0.17)	0.81 (0.18)	**0.001**
LVEDV (ml)	80.40 (26.18)	59.90 (21.12)	**<0.001**
LVESV (ml)	42.60 (18.68)	30.82 (15.01)	**<0.001**
SV (ml)	37.79 (13.38)	29.22 (10.94)	**<0.001**
EF (%)	47.87 (10.81)	49.14 (11.54)	0.110
LA diameter (mm)	44.75 (5.88)	41.94 (5.86)	**<0.001**
LAA (cm^2^)	26.31 (5.62)	24.54 (5.01)	**<0.001**
RAA (cm^2^)	24.84 (6.39)	21.05 (5.98)	**<0.001**
E/A	2.14 (1.10)	1.99 (1.04)	0.086
E/e′ average	16.82 (6.27)	20.33 (8.04)	**<0.001**
MAPSE (mm)	8.00 (2.52)	7.93 (2.51)	0.727
TAPSE (mm)	15.12 (4.86)	14.94 (4.88)	0.696
RV S′ velocity (cm/s)	10.12 (3.05)	10.08 (3.25)	0.915
PASP (mmHg)	37.50 (14.04)	37.37 (15.87)	0.910
TAPSE/PASP (mm/mmHg)	0.49 (0.59)	0.48 (0.57)	0.809
GLS (%)	−10.73 (3.60)	−11.27 (3.94)	**0.043**
Significant MR	175 (11.78%)	48 (19.4%)	**0.001**
Significant TR	249 (16.77%)	59 (23.89%)	**0.006**

Data are presented as mean (standard deviation), or *n* (%).

EF, ejection fraction; GLS, global longitudinal strain; IVSd, interventricular septum thickness in diastole; LA, left atrium; LAA, left atrial area; LVEDD, left ventricular end‐diastolic diameter; LVEDV, left ventricular end‐diastolic volume; LVESD, left ventricular end‐systolic diameter; LVESV, left ventricular end‐systolic volume; LVM, left ventricular mass; MAPSE, mitral annular plane systolic excursion; MR, mitral regurgitation; MWT, mean wall thickness; PASP, pulmonary artery systolic pressure; PWTd, posterior wall thickness in diastole; RAA, right atrial area; RV S′, right ventricle systolic excursion; RWT, relative wall thickness; SV, stroke volume; TAPSE, tricuspid annular plane systolic excursion; TR, tricuspid regurgitation.

All *p*‐values are adjusted for age.

**Figure 2:**
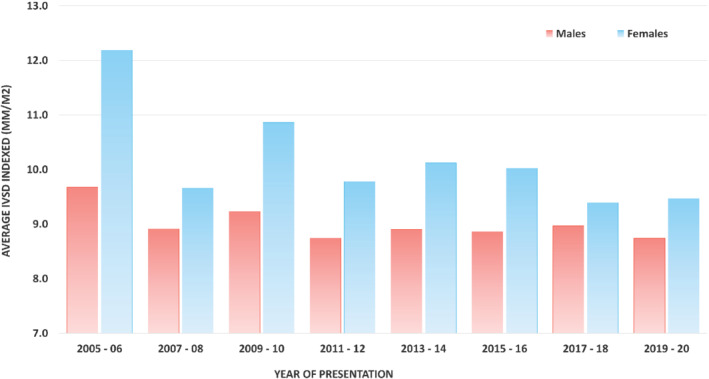
Average interventricular septum in diastole (IVSd) indexed to body surface area in males and females at presentation from 2005 to 2020. Patients divided into groups of 2‐year blocks. Patients presenting between 2000 and 2004 were not included due to limited patient numbers. Patients data from 1 January 2021 to censor date were not included as this represents only an 8‐month period of time.

However, when values were indexed for BSA (*Table* *3*), female patients were found to have a significantly greater mean IVSd (9.62 vs. 8.88 mm/m^2^, *p* < 0.001), posterior wall thickness in diastole (PWTd) and mean wall thickness (MWT) than men across the whole cohort. When indexed to height, mean PWTd remained significantly greater in females but mean IVSd was similar between sexes. LVM remained significantly elevated in males than in females when indexed to BSA or height. *Figure* *2* shows mean IVSd indexed to BSA at presentation in both men and women based on their year of presentation. Mean IVSd indexed to BSA was fairly constant in males throughout the study period but in females a tendency to decrease over the same study period was noted. Online supplementary *Figure* *S1* presents the proportion of males and females within tertiles of both non‐indexed and indexed IVSd. When non‐indexed, 43.9% of females were within the lowest tertile but when indexed to either BSA or height, 51.6% and 42% of females were within the highest tertile, respectively. In comparison, the distribution of males remained similar across all tertiles irrespective of whether IVSd was indexed or not.

**Table 3:** Echocardiographic findings at baseline indexed to body surface area and height in patients with transthyretin amyloid cardiomyopathy

Variables	All patients (*n* = 1732)		
	Male (*n* = 1485)	Female (*n* = 247)	*p*‐value
IVSd indexed to BSA (mm/m^2^)	8.88 (1.55)	9.62 (2.11)	**<0.001**
IVSd indexed to height (mm/m)	9.84 (1.68)	10.05 (2.14)	0.154
PWTd indexed to BSA (mm/m^2^)	8.57 (1.58)	9.47 (1.96)	**<0.001**
PWTd indexed to height (mm/m)	9.50 (1.73)	9.89 (2.04)	**0.007**
LVM index to BSA (g/m^2^)	165.13 (41.69)	154.66 (45.71)	**0.001**
LVM indexed to height (mm/m)	183.24 (47.52)	161.61 (50.14)	**<0.001**
LVEDD indexed to BSA (mm/m^2^)	22.64 (3.70)	23.71 (4.45)	**<0.001**
LVESD indexed to BSA (mm/m^2^)	17.22 (3.64)	17.63 (4.15)	0.119
MWT indexed to BSA (mm/m^2^)	8.73 (1.52)	9.55 (1.99)	**<0.001**
LVEDV indexed to BSA (ml/m^2^)	41.05 (13.68)	35.07 (13.08)	**<0.001**
LVESV indexed to BSA (ml/m^2^)	21.74 (9.71)	17.95 (9.06)	**<0.001**
SV indexed to BSA (ml/m^2^)	19.33 (6.90)	17.20 (6.87)	**<0.001**
LAA indexed to BSA (cm^2^/m^2^)	13.52 (3.32)	14.55 (3.71)	**<0.001**
RAA indexed to BSA (cm^2^/m^2^)	12.73 (3.57)	12.44 (3.92)	0.173

Data are presented as mean (standard deviation).

BSA, body surface area; IVSd, interventricular septum thickness in diastole; LAA, left atrial area; LVEDD, left ventricular end‐diastolic diameter; LVEDV, left ventricular end‐diastolic volume; LVESD, left ventricular end‐systolic diameter; LVESV, left ventricular end‐systolic volume; LVM, left ventricular mass; MWT, mean wall thickness; PWTd, posterior wall thickness in diastole; RAA, right atrial area; SV, stroke volume.

All *p*‐values are adjusted for age.

When split by genotype (online supplementary *Table* *S2*), a significant difference in wall thickness parameters indexed to BSA between males and females was obtained across all genotypes with the exception of IVSd index in wtATTR‐CM. When indexed to height, no significant difference was observed in wall thickness parameters across all genotypes. A significant difference in LVM when indexed to height between males and females was observed across all genotypes. When indexed to BSA, LVM was significantly different only in patients with wtATTR‐CM. LVEDD and left atrial area indexed to BSA were also significantly greater in females compared to males across the whole cohort of patients (*Table* *3*) but did not remain significant when divided into certain genotypes (online supplementary *Table* *S2*). Mean right atrial area indexed to BSA was only significantly different in the T60A‐hATTR‐CM cohort. Multivariable linear regression analysis demonstrated that sex was an independent predictor of IVSd when indexed to either height (online supplementary *Table* *SA*) or BSA (online supplementary *Table* *SB*) and after being adjusted for genotype.

Volumetric parameters such as LVEDV and LVESV demonstrated smaller mean LV cavity sizes for females when indexed to BSA across the entire cohort (*Table* *3*) and when split by genotype with the exception of the T60A‐ATTR‐CM cohort (online supplementary *Table* *S2*). SV indexed to BSA remained significantly smaller on average for females across the entire cohort (*Table* *3*) and also in the wtATTR‐CM group (online supplementary *Table* *S2*).

When analysing the cohort as a whole (*Table* *2*), mean LV ejection fraction was similar between males and females, whereas global longitudinal strain (GLS) was significantly better in females than males. No differences were found between sexes in mean mitral and tricuspid annular plane systolic excursion and right ventricular systolic function. In terms of diastolic function, there was no significant difference in mean E/A ratio between sexes but mean E/e′ was significantly greater in females than males. Finally, there was a greater degree of significant mitral and tricuspid regurgitation in females compared to males across the whole cohort. A total of 114 patients (6.6%) were on TTR‐specific therapies (92 males and 22 females). Given the limited number of patients in these cohorts, sub‐analysis of these patients was not performed, as we would be unable to draw definite conclusions.

### Disease progression between men and women in each genotype

A total of 906 patients had a repeat echocardiogram at 1 year. A multivariable linear regression analysis compared mean values of each echocardiographic variable at 1 year in males and females, after adjusting for the baseline value of the variable and age. Results are shown in online supplementary *Table* *S3*. The vast majority of parameters showed similar progression of disease in females compared to males across the entire cohort. The few differences in echocardiographic parameters indicated greater disease progression in females. Volumetric parameters including LVEDV, LVESV and SV (all indexed to BSA) were significantly lower in females at 12 months. IVSd, PWTd and MWT (all indexed to BSA) were significantly higher in females at 12 months. Mean values of tricuspid regurgitation and pulmonary artery systolic pressure were also significantly higher in females at 12 months.

Of the 906 patients with a repeat echocardiogram at 1 year, 595 patients were wtATTR‐CM, 99 were T60A‐ATTR‐CM and 212 were V122I‐ATTR‐CM. In the wtATTR‐CM cohort, only LVEDV and LVESV (both indexed to BSA) had significantly different means at 12 months with mean values in males greater than in females. In the T60A‐ATTR‐CM cohort, mean IVSd indexed to BSA was significantly greater in females and mean E/e′ greater in males at 12 months. In the V122I‐ATTR‐CM cohort, mean GLS was significantly greater in males than females at 12 months.

### Sex and prognosis

At a mean follow‐up of 37 (SD 30) months, 800 (46.2%) of 1732 patients had died, including 464 (42.4%) with wtATTR‐CM, 238 (55.2%) with V122I‐hATTR‐CM and 98 (47.6%) with T60A‐hATTR‐CM.

When classified by sex, 684 (46%) of 1485 males and 116 (47%) of 247 females died. In the wtATTR‐CM group, 22 (33.3%) of the 66 females died compared to 442 (43%) of the 1029 males. In the T60A‐hATTR‐CM group, 33 (54.1%) of the 61 females died compared to 65 (44.8%) of the 145 males. In the V122I‐hATTR‐CM group, 61 (50.8%) of the 120 females died compared to 177 (56.9%) of the 311 males.

**Figure 3:**
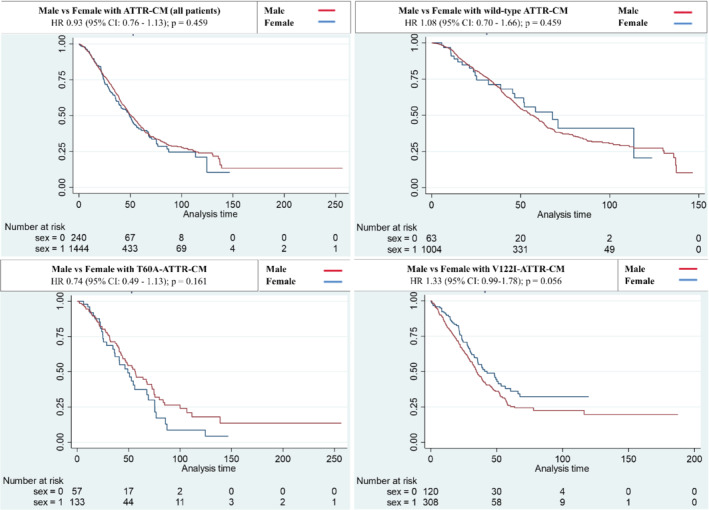
Kaplan–Meier survival curves comparing males and females, divided by genotype. Kaplan–Meier curves displaying the prognostic impact of sex on survival in patients with transthyretin amyloid cardiomyopathy (ATTR‐CM) when separated for each genotype: all patients (top left), wild‐type (wt)ATTR‐CM (top right), T60A‐ATTR‐CM (bottom left), V122I‐ATTR‐CM (bottom right). Analysis time (in months) on horizontal axis and cumulative survival probability on vertical axis. CI, confidence interval; HR, hazard ratio.

Kaplan–Meier survival curves are shown in *Figure* *3*. There was no significant difference in survival between men and women across the whole cohort or when split by genotype and adjusted for age. Median survival in wtATTR‐CM was 57.0 months in males and 67.8 months in females. Median survival in T60A‐hATTR‐CM was 56.5 months in males compared to 49.2 months in females. Median survival in V122I‐hATTR‐CM was 34.6 months in males compared to 42.8 months in females.

Comparison of the risk of death between males and females based on the degree of progression of echocardiographic variables from baseline to 1 year is reported in online supplementary *Table* *S4*. There was no difference in risk of death when comparing males with females for all echocardiographic variables studied, either in the whole cohort or when divided by genotype.

## Discussion

This is the largest study focused on sex differences in a large, well‐characterized cohort of patients with cardiac ATTR‐CM. The main findings of this study are: (i) female prevalence was greater in hATTR‐CM compared to wtATTR‐CM, with no evidence of a difference between the two hereditary genotypes studied; (ii) across all genotypes, females were older on average at presentation, with an age difference of approximately 3 years between sexes; (iii) body size significantly influenced the assessment of disease severity, demonstrated by the observation that the overall structural and functional phenotype was similar between sexes when indexed to body size, with the only significant differences pointing towards a mildly worse phenotype in females; (iv) the absence of significant differences in the clinical phenotype were also confirmed when disease progression was assessed by follow‐up echocardiography after 1 year, at which point no significant differences were noted; and (v) there was no significant difference between males and females in mortality in the overall population and when divided by genotype after adjusting for age at presentation (*Graphical Abstract*).

Historically, most studies assessing structural and functional differences between sexes in patients with ATTR‐CM used non‐indexed echocardiographic values, reporting lower wall thickness, LVM and smaller atrial size in females which has engendered the notion that females with ATTR‐CM have a less severe disease phenotype compared to males. Our study shows that after indexing for body size, functional and structural phenotypes are broadly very similar, with females actually showing a slightly worse phenotype in some respects compared to men at presentation.

Having suspicion of cardiac amyloidosis (CA) is an essential step towards the correct diagnosis. Echocardiography is the first imaging modality used in all patients with heart failure symptoms, and increased wall thickness on echocardiography remains the most critical finding to raise the initial suspicion of CA. Current guidelines recommend a value for non‐indexed IVSd of 12 mm or greater on echocardiography to prompt referral for investigation for CA. Indexing for body size is recommended in certain conditions due to different thresholds related to various characteristics, but this is not the case in CA. The above threshold does not take body size into account and has therefore been persistently applied to both males and females both for the diagnosis of patients with suspected CA, and its application for criteria for inclusion in clinical trials. Importantly, both normal and abnormal anatomy is typically smaller in women compared to men, a finding which our study demonstrates is true for patients with ATTR‐CM. Therefore, the use of this single, non‐indexed value for wall thickness in ATTR‐CM is highly likely to contribute to under‐diagnoses of the disease among females, who are less likely to meet the diagnostic wall thickness threshold in comparison to their male counterparts.

The prevalence of males compared to females remains higher across all genotypes, something observed also in previous literature and possible theories have been hypothesized. Firstly, serum TTR concentration can be affected by circulating hormones, with oestrogens having been shown to reduce serum TTR concentration and androgens increase them.^23^ One study found that women with hATTR‐CM may be less severely affected by the disease before the menopause and hypothesized a possible, but largely unexplored, role of female sex hormones being protective against disease manifestation.^24^ The potential sex bias of using non‐indexed echocardiographic parameters in diagnostic algorithms may also contribute to the observed difference in prevalence between men and women. This hypothesis is also supported by several studies screening for wtATTR‐CM in various populations, which did not find a significant male predominance.^12^, ^25^, ^26^ Further subgroup analysis offers more insight into this. Both hereditary forms of ATTR‐CM studied have a male prevalence of 70–73% compared to a much higher 94% in wtATTR‐CM. The impact of early screening within families affected by hATTR‐CM is likely to identify more females with cardiac disease and the values observed in our study for these genotypes are likely to be more representative of the true sex prevalence. Overall, these results suggest that ATTR‐CM may be more common in females than currently thought and that the aforementioned deficiencies of the existing diagnostic algorithms have contributed to significant under‐diagnosis in women. Unfortunately, eligibility based on a non‐indexed single cut‐off value for wall thickness has been used in all recent major clinical trials investigating new therapeutic approaches in CA, raising the strong possibility that women have been under‐represented in these studies. In addition, our study does not consider patients with hATTR who have a predominantly non‐cardiac phenotype. Previous studies have shown that the male imbalance is less evident in the aforementioned cohort,^9^ which raises the possibility that the presence of a cardiac phenotype may be a potential contributing factor for the observed male imbalance.

Interestingly, throughout the two‐decade study period during which our study patients had been diagnosed with ATTR‐CM at our centre, mean indexed IVSd at presentation remained fairly constant year by year in males but progressively decreased in women, potentially explained by an increasing awareness of the disease in females. In addition, those females whose diagnoses were prompted by the presence of an LV wall thickness greater than 12 mm are likely to be further along the course of the disease compared with their male counterparts. This would fit with our findings that females are on average 3 years older at presentation at which time indexed values of wall thickness were similar or greater in men. The mildly worse clinical phenotype at presentation was also supported by other structural and functional markers, with greater degree of diastolic failure and more severe degree of mitral and tricuspid regurgitation.

The absence of significant differences in the clinical phenotype was also confirmed when disease progression was assessed with an echocardiogram repeated after 1 year: there were no differences in the vast majority of parameters reflecting disease progression in females compared to males in the cohort of 906 patients in whom echocardiographic follow‐up was possible using a broad range of deformation and non‐deformation parameters. These findings do not support the concept of a less aggressive disease trajectory in women, as when appropriately indexed, the only differences found in some of the parameters were in keeping with greater disease progression in females compared to males. Finally, when prognosis was assessed, there was no significant difference in survival between men and women across the whole cohort and when split by genotype.

## Conclusion

This is the largest study to date focused on sex differences in a large, well‐characterized cohort of patients with ATTR‐CM. Our finding, contrary to previous dogmas, suggest that there are no differences in the clinical phenotype between male and female sex in ATTR‐CM, both in the overall population and when analysed by genotype. This is supported by an extensive array of echocardiographic data which included a variety of deformation and non‐deformation parameters that were comparable between men and women when taking body size into account; this impression was further reinforced by the absence of significant differences in both disease progression on serial echocardiography and overall mortality. The analysis highlighted the deficiencies in using non‐indexed values, which can not only lead to the inaccurate perception of a milder clinical phenotype in women compared to men, but have been shown to result in female patients presenting at an older age and with a worse phenotype compared to men. We conclude this study by highlighting the urgent need for the revision of existing recommendations, clinical guidelines and inclusion criteria in clinical studies, which currently adopt a single non‐indexed threshold for wall thickness for the diagnosis of CA but have inadvertently resulted in females being both under‐diagnosed and presenting at a later disease stage compared to males.

### Limitations

This study was limited to the analysis of the three most common genotypes of ATTR‐CM. It would be important to study other well‐characterized pathogenic mutations of the *TTR* gene which are implicated in ATTR amyloidosis with a predominant polyneuropathy phenotype. Furthermore, we did not include patients that were genetic carriers for hATTR. Analysis of these cohorts of patients is likely to offer further insight into the natural history of the disease. Secondly, there is a potential for selection bias as our study cohort included only those patients who had a confirmed diagnosis of ATTR‐CM and the overwhelming majority was noted as having a non‐indexed wall thickness of >12 mm. We would advocate wider screening studies of males and females prior to a diagnosis being established in order to corroborate our observations from this study.

### Funding

M. Fontana is supported by a British Heart Foundation Intermediate Clinical Research Fellowship (FS/18/21/33447).

**Conflict of interest:** none declared.

## Supporting information


**Appendix S1**. Supporting Information
