# Supplementary material for: Sex differences among patients with transthyretin amyloid cardiomyopathy – from diagnosis to prognosis
Source: Eur J Heart Fail. 2022 Aug 16;24(12):2355–63. doi: 10.1002/ejhf.2646 (PMC10087683; doi:10.1002/ejhf.2646)

**Supplementary Figure Legends**

**Supplementary Figure 1 – Proportion of males and females within tertiles of both non-indexed and indexed interventricular septum in diastole (IVSd)**

Patients grouped into tertiles based on (a) IVSd when non-indexed, (b) IVSd indexed to body surface area (BSA) and (c) IVSd indexed to height and separated into males and females to determine the relative proportion of patients within each tertile.

**Supplementary Table 1 – Echocardiographic findings at baseline in patients with ATTR-CM classified by genotype & subdivided by sex**

| Variables | Wild-type (n = 1095) | | | T60A (n = 206) | | | V122I (n = 431) | | |
| --- | --- | --- | --- | --- | --- | --- | --- | --- | --- |
|  | **Male**  **(n = 1029)** | **Female (n = 66)** | **P-value** | **Male**  **(n = 145)** | **Female (n = 61)** | **P-value** | **Male**  **(n = 311)** | **Female**  **(n = 120)** | **P-value** |
| IVSd (mm) | **17.16 (2.34)** | **15.98 (2.44)** | **0.001** | **17.14 (2.76)** | **16.09 (3.30)** | **0.007** | **17.05 (2.16)** | **16.26 (2.41)** | **0.003** |
| PWTd (mm) | 16.50 (2.44) | 16.02 (2.39) | 0.150 | 16.66 (2.79) | 15.75 (3.33) | 0.075 | **16.60 (2.38)** | **15.95 (2.14)** | **0.007** |
| LVM (g) | **320.11 (83.19)** | **240.67 (90.94)** | **<0.001** | **324.24 (92.01)** | **252.50 (86.71)** | **<0.001** | **305.52 (82.30)** | **263.92 (69.99)** | **<0.001** |
| LVEDD (mm) | **44.04 (5.70)** | **38.89 (4.96)** | **<0.001** | **43.91 (5.78)** | **40.07 (4.84)** | **<0.001** | **42.74 (6.19)** | **40.29 (5.70)** | **<0.002** |
| LVESD (mm) | **33.35 (6.15)** | **28.69 (5.12)** | **<0.001** | 31.40 (6.20) | 29.68 (5.38) | 0.051 | **33.60 (6.64)** | **30.50 (5.56)** | **<0.001** |
| MWT (mm) | **16.66 (2.78)** | **15.19 (4.23)** | **0.001** | **16.43 (3.35)** | **15.65 (3.85)** | **0.011** | **16.66 (2.68)** | **15.97 (2.60)** | **0.027** |
| RWT | **0.77 (0.17)** | **0.84 (0.16)** | **0.008** | 0.77 (0.18) | 0.80 (0.22) | 0.939 | 0.80 (0.19) | 0.81 (0.17) | 0.653 |
| LVEDV (ml) | **80.75 (24.86)** | **52.35 (16.59)** | **<0.001** | **82.33 (28.13)** | **64.48 (21.43)** | **0.002** | **78.48 (29.26)** | **61.54 (22.14)** | **<0.001** |
| LVESV (ml) | **41.67 (17.10)** | **24.80 (11.59)** | **<0.001** | **41.26 (18.32)** | **30.72 (12.56)** | **<0.001** | **46.14 (22.79)** | **33.72 (16.59)** | **<0.001** |
| SV (ml) | **39.08 (13.15)** | **28.00 (8.87)** | **<0.001** | **41.05 (14.94)** | **33.76 (14.03)** | **0.046** | **32.40 (11.95)** | **27.82 (9.80)** | **0.003** |
| EF (%) | **49.04 (10.28)** | **53.07 (10.71)** | **0.011** | 51.35 (9.78) | 51.44 (10.65) | 0.655 | **42.77 (11.23)** | **46.32 (11.60)** | **0.004** |
| LA diameter (mm) | **45.37 (5.79)** | **42.79 (5.30)** | **0.005** | **42.03 (5.99)** | **39.21 (5.96)** | **0.002** | 43.82 (5.72) | 42.76 (5.75) | 0.197 |
| LAA (cm^2^) | 26.72 (5.68) | 25.22 (4.61) | 0.069 | **23.88 (5.01)** | **21.63 (4.57)** | **0.001** | 26.00 (5.39) | 25.54 (4.92) | 0.565 |
| RAA (cm^2^) | **25.15 (6.30)** | **22.09 (6.85)** | **<0.001** | **21.45 (5.10)** | **16.68 (3.77)** | **<0.001** | **25.26 (6.78)** | **22.51 (5.41)** | **<0.001** |
| E/A | 2.09 (1.10) | 1.85 (1.05) | 0.307 | 1.62 (0.97) | 1.59 (0.90) | 0.892 | 2.49 (1.05) | 2.26 (1.04) | 0.071 |
| E/e’ average | **16.72 (6.28)** | **21.54 (8.81)** | **<0.001** | 16.93 (7.28) | 21.07 (8.72) | 0.062 | **17.10 (5.79)** | **19.39 (7.26)** | **0.002** |
| MAPSE (mm) | 8.14 (2.56) | 8.34 (2.50) | 0.654 | 8.44 (2.55) | 8.22 (2.59) | 0.938 | 7.38 (2.28) | 7.62 (2.46) | 0.330 |
| TAPSE (mm) | 15.09 (4.91) | 14.79 (5.60) | 0.759 | 16.97 (4.69) | 15.97 (4.16) | 0.806 | 14.48 (4.59) | 14.56 (4.78) | 0.619 |
| RV S’ (cm/s) | 10.21 (3.13) | 10.42 (3.86) | 0.423 | 10.61 (2.93) | 10.05 (2.85) | 0.725 | 9.66 (2.79) | 9.91 (3.08) | 0.289 |
| PASP (mmHg) | **37.57 (12.99)** | **34.36 (16.20)** | **0.043** | 30.17 (16.63) | 30.65 (17.58) | 0.687 | 39.96 (15.25) | 42.21 (13.15) | 0.304 |
| TAPSE/PASP | 0.66 (0.61) | 0.59 (0.73) | 0.613 | 0.66 (0.61) | 0.59 (0.73) | 0.613 | 0.43 (0.50) | 0.39 (0.41) | 0.400 |
| GLS (%) | -11.04 (3.63) | -11.65 (4.13) | 0.252 | -11.63 (3.82) | -12.20 (3.89) | 0.114 | **-9.35 (3.06)** | **-10.67 (3.80)** | **0.001** |
| Significant MR | 102 (10.0%) | 8 (12.1%) | 0.584 | 19 (13.1%) | 6 (9.8%) | 0.509 | 67 (21.5%) | 34 (28.3%) | 0.135 |
| Significant TR | 140 (13.7%) | 14 (21.1%) | 0.095 | 9 (6.2%) | 8 (13.1%) | 0.101 | 100 (32.2%) | 37 (30.8%) | 0.780 |

All P-values are adjusted for age. Data are presented as means (SD), with the exception of significant MR and TR which are presented as number (%). E/A – mitral inflow E/A ratio, EF – ejection fraction, GLS – global longitudinal strain, IVSd – interventricular systolic wall thickness in diastole, LA – left atrium, LAA – left atrial area, LVEDD – left ventricular end diastolic diameter, LVEDV – left ventricular end diastolic volume, LVESD – left ventricular end systolic diameter, LVESV – left ventricular end systolic volume, LVM – left ventricular mass, MAPSE – mitral annular plane systolic excursion, MR – mitral regurgitation, MWT – mean wall thickness, PASP – pulmonary artery systolic pressure, PWTd – posterior wall thickness in diastole, RAA – right atrial area, RV S’ velocity – right ventricle systolic excursion velocity, SV – stroke volume, TAPSE – tricuspid annular plane systolic excursion, TAPSE/PASP – tricuspid annular plane systolic excursion to pulmonary artery systolic pressure ratio, TR – tricuspid regurgitation

**Supplementary Table 2 – Echocardiographic findings at baseline indexed to body surface area and height, in patients with ATTR-CM classified by genotype and subdivided by sex**

| Variables | Wild-type (n = 1095) | | | T60A (n = 206) | | | V122I (n = 431) | | |
| --- | --- | --- | --- | --- | --- | --- | --- | --- | --- |
|  | **Male (n = 1029)** | **Female (n = 66)** | **P-value** | **Male (n = 145)** | **Female (n = 61)** | **P-value** | **Male (n = 311)** | **Female (n = 120)** | **P-value** |
| IVSd index to BSA (mm/m2) | 8.87 (1.53) | 9.28 (2.37) | 0.172 | **8.72 (2.03)** | **10.10 (2.15)** | **0.001** | **8.96 (1.35)** | **9.56 (1.92)** | **0.002** |
| IVSd indexed for height (mm/m) | 9.67 (1.66) | 9.90 (2.41) | 0.910 | 9.49 (2.10) | 9.98 (2.03) | 0.153 | 9.89 (1.53) | 10.16 (2.06) | 0.209 |
| PWTd index to BSA (mm/m2) | **8.53 (1.54)** | **9.32 (2.33)** | **0.001** | **8.50 (2.05)** | **9.84 (1.98)** | **0.002** | **8.73 (1.45)** | **9.37 (1.72)** | **0.001** |
| PWTd indexed for height (mm/m) | 9.49 (1.69) | 9.91 (2.40) | 0.193 | 9.27 (2.14) | 9.72 (1.92) | 0.164 | 9.63 (1.65) | 9.96 (1.91) | 0.102 |
| LVM index to BSA (g/m2) | **166.40 (41.75)** | **146.73 (49.63)** | **0.004** | 166.90 (47.47) | 162.23 (50.91) | 0.568 | 161.03 (37.61) | 155.18 (40.64) | 0.182 |
| LVM indexed for height (mm/m) | **185.13 (46.65)** | **157.39 (53.85)** | **<0.001** | **180.08 (56.77)** | **156.40 (7.59)** | **0.011** | **178.31 (45.57)** | **166.09 (45.91)** | **0.016** |
| LVEDD index to BSA (mm/m^2^) | 22.75 (3.51) | 22.61 (5.20) | 0.807 | **22.53 (4.79)** | **25.31 (3.53)** | **<0.001** | **22.35 (3.72)** | **23.52 (4.25)** | **0.010** |
| LVESD index to BSA (mm/m2) | 17.21 (3.46) | 16.61 (4.34) | 0.247 | **16.51 (4.42)** | **18.79 (3.78)** | **0.001** | 17.54 (3.80) | 17.59 (4.12) | 0.998 |
| MWT index to BSA (mm/m2) | **8.70 (1.46)** | **9.31 (2.32)** | **0.010** | **8.69 (2.22)** | **9.97 (2.03)** | **0.005** | **8.84 (1.33)** | **9.47 (1.76)** | **0.001** |
| MWT indexed for height (mm/m) | 9.69 (1.56) | 9.91 (2.38) | 0.248 | 9.38 (2.10) | 9.85 (1.94) | 0.552 | 9.76 (1.51) | 10.06 (1.93) | 0.113 |
| LVEDV index to BSA (ml/m^2^) | **41.19 (13.06)** | **30.26 (11.20)** | **<0.001** | 41.06 (15.77) | 40.32 (12.30) | 0.855 | **40.61 (14.69)** | **35.17 (13.47)** | **0.002** |
| LVESV index to BSA (ml/m^2^) | **21.22 (8.89)** | **14.30 (7.37)** | **<0.001** | 20.66 (9.96) | 19.34 (8.10) | 0.546 | **23.85 (11.66)** | **19.14 (9.77)** | **<0.001** |
| SV index to BSA (ml/m^2^) | **19.97 (6.82)** | **16.21 (5.98)** | **<0.001** | 20.56 (7.98) | 20.97 (7.85) | 0.335 | 16.79 (6.03) | 16.04 (6.27) | 0.456 |
| LAA index to BSA (cm^2^/m^2^) | 13.78 (3.14) | 14.70 (4.06) | 0.081 | 12.23 (3.27) | 13.60 (2.75) | 0.114 | **13.22 (3.75)** | **14.90 (3.86)** | **<0.001** |
| RAA index to BSA (cm^2^/m^2^) | 12.93 (3.35) | 12.89 (4.85) | 0.523 | **10.96 (3.01)** | **10.47 (2.23)** | **0.046** | 12.81 (4.22) | 13.09 (3.74) | 0.880 |

All P-values are adjusted for age. Data are presented as means (and standard deviation). BSA – body surface area, IVSd – interventricular systolic wall thickness in diastole, LAA – left atrial area, LVEDD – left ventricular end diastolic diameter, LVEDV – left ventricular end diastolic volume, LVESD – left ventricular end systolic diameter, LVESV – left ventricular end systolic volume, MWT – mean wall thickness, PWTd – posterior wall thickness in diastole, RAA – right atrial area, SV – stroke volume

**Supplementary Table 3 – Regression coefficient representing the difference in means (males minus females) of echocardiographic variable at 1 year**

|  | All patients (n=906; 116 females) | | Wild-type (n=595; 26 females) | | T60A (n=99; 29 females) | | V122I (n=212; 61 females) | |
| --- | --- | --- | --- | --- | --- | --- | --- | --- |
| Echocardiographic Variable | **Regression Coefficient**  **(95% CI)** | **P-value** | **Regression Coefficient**  **(95% CI)** | **P-value** | **Regression Coefficient**  **(95% CI)** | **P-value** | **Regression Coefficient**  **(95% CI)** | **P-value** |
| IVSd index to BSA | **-0.20 (-0.33; -0.06)** | **<0.01** | -0.01 (-0.25; 0.22) | 0.92 | **-0.72 (-1.29; -0.14)** | **0.02** | -0.09 (-0.26; 0.09) | 0.34 |
| PWTd index to BSA | **-0.23 (-0.39; -0.06)** | **<0.01** | -0.17 (-0.48; 0.13) | 0.27 | -0.45 (-1.03; 0.14) | 0.14 | -0.12 (-0.35; 0.11) | 0.31 |
| MWT index to BSA | **-0.17 (-0.32; -0.02)** | **0.03** | -0.05 (-0.34; 0.23) | 0.71 | -0.56 (-1.11; 0.002) | 0.05 | -0.06 (-0.22; 0.10) | 0.45 |
| RWT | -0.01 (-0.03; 0.01) | 0.40 | 0 (-0.41; 0.42) | 0.98 | -0.01 (-0.06; 0.05) | 0.84 | -0.01 (-0.04; 0.03) | 0.71 |
| LVEDD index to BSA | -0.33 (-0.77; 0.10) | 0.13 | -0.40 (-1.20; 0.41) | 0.34 | -0.91 (-2.46; 0.63) | 0.24 | -0.22 (-0.83; 0.40) | 0.49 |
| LVESD index to BSA | -0.38 (-0.88; 0.12) | 0.13 | 0.04 (-0.96; 1.04) | 0.94 | -0.63 (-2.20; 0.93) | 0.42 | -0.41 (-1.07; 0.25) | 0.22 |
| LVEDV index to BSA | **3.37 (1.12; 5.63)** | **<0.01** | **4.43 (0.003; 8.86)** | **0.05** | 0.52 (-5.76; 6.79) | 0.87 | 0.84 (-2.33; 4.01) | 0.60 |
| LVESV index to BSA | **1.65 (0.22; 3.07)** | **0.02** | **3.15 (0.44; 5.86)** | **0.02** | 0.13 (-3.38; 3.63) | 0.94 | 0.70 (-1.74; 3.13) | 0.57 |
| SV index to BSA | **1.69 (0.42; 2.95)** | **<0.01** | 1.65 (-0.90; 4.21) | 0.21 | 1.73 (-1.56; 5.03) | 0.30 | -0.31 (-1.83; 1.20) | 0.69 |
| EF (%) | -0.81 (-2.58; 0.96) | 0.37 | -3.44 (-6.95; 0.06) | 0.05 | 0.90 (-3.58; 5.38) | 0.69 | -2.00 (-4.74; 0.73) | 0.15 |
| LAD | **2.23 (1.29; 3.16)** | **<0.001** | 0.85 (-0.99; 2.70) | 0.36 | **3.28 (0.83; 5.74)** | **<0.01** | **2.23 (0.80; 3.65)** | **0.002** |
| LAA 4ch index to BSA | -0.24 (-0.71; 0.22) | 0.30 | -0.69 (-1.60; 0.21) | 0.13 | 0.54 (-0.69; 1.76) | 0.39 | -0.49 (-1.19; 0.21) | 0.17 |
| RAA 4ch index to BSA | 0.14 (-0.35; 0.63) | 0.57 | 0.54 (-0.42; 1.50) | 0.27 | 0.18 (-0.78; 1.14) | 0.72 | 0.37 (-0.49; 1.23) | 0.40 |
| E/A Ratio | -0.01 (-0.23; 0.22) | 0.96 | 0.16 (-0.27; 0.60) | 0.46 | 0.26 (-0.30; 0.82) | 0.35 | -0.17 (-0.50; 0.15) | 0.30 |
| E/e’ average | -0.59 (-1.69; 0.50) | 0.29 | -1.49 (-3.71; 0.74) | 0.19 | **3.33 (0.49; 6.17)** | **0.02** | -1.07 (-2.48; 0.34) | 0.14 |
| MAPSE | 0.40 (-0.06; 0.86) | 0.09 | 0.10 (-0.78; 0.98) | 0.82 | 0.58 (-0.64; 1.80) | 0.35 | -0.11 (-0.81; 0.58) | 0.75 |
| TAPSE | -0.06 (-0.84; 0.71) | 0.88 | -0.76 (-2.24; 0.71) | 0.31 | 1.02 (-0.87; 2.91) | 0.29 | -0.42 (-1.70; 0.85) | 0.51 |
| S’ tricuspid | 0.003 (-0.53; 0.54) | 0.99 | 0.02 (-1.02; 1.06) | 0.97 | -0.13 (-1.49; 1.23) | 0.85 | -0.27 (-1.05; 0.51) | 0.50 |
| TR Gradient | **-2.49 (-4.40; -0.49)** | **0.02** | -3.39 (-7.26; 0.48) | 0.09 | -1.92 (-6.19; 2.36) | 0.37 | -2.44 (-5.91; 1.04) | 0.17 |
| PASP | **-2.89 (-5.26; -0.52)** | **0.02** | -3.53 (-8.27; 1.20) | 0.14 | -1.94 (-6.59; 2.72) | 0.41 | -2.40 (-6.45; 1.66) | 0.25 |
| GLS | 0.25 (-0.35; 0.85) | 0.41 | 0.34 (-0.89; 1.57) | 0.59 | -0.48 (-1.90; 0.93) | 0.50 | **1.20 (0.39; 2.02)** | **0.004** |

Each regression coefficient, derived from multivariable linear regression analysis is adjusted for the baseline value of the variable and age. Sex is coded as male = 1 and females = 0 so that positive regression coefficient indicates that at 1 year, the mean value of the relevant variable was higher in males than in females. Negative regression coefficient indicates that at 1 year, the mean value of the relevant variable was higher in females than in males. BSA – body surface area, E/A – mitral inflow E/A ratio, EF – ejection fraction, GLS – global longitudinal strain, IVSd – interventricular systolic wall thickness in diastole, LAD – left atrial diameter, LAA – left atrial area, LVEDD – left ventricular end diastolic diameter, LVEDV – left ventricular end diastolic volume, LVESD – left ventricular end systolic diameter, LVESV – left ventricular end systolic volume, MAPSE – mitral annular plane systolic excursion, MR – mitral regurgitation, MWT – mean wall thickness, PASP – pulmonary artery systolic pressure, PWTd – posterior wall thickness in diastole, RAA – right atrial area, RV S’ velocity – right ventricle systolic excursion velocity, SV – stroke volume, TAPSE – tricuspid annular plane systolic excursion, TAPSE/PASP – tricuspid annular plane systolic excursion to pulmonary artery systolic pressure ratio, TR – tricuspid regurgitation

**Supplementary Table 4 – Hazard ratio (HR) with 95% confidence interval (CI) comparing the hazard of death in males and females**

|  | All patients (n=906; 116 females) | | Wild-type (n=595; 26 females) | | T60A (n=99; 29 females) | | V122I (n=212; 61 females) | |
| --- | --- | --- | --- | --- | --- | --- | --- | --- |
| Echocardiographic variable as covariate | **HR (95% CI)** | **P-value** | **HR (95% CI)** | **P-value** | **HR (95% CI)** | **P-value** | **HR (95% CI)** | **P-value** |
| IVSd index to BSA | 0.91 (0.70-1.17) | 0.45 | 1.10 (0.64-1.87) | 0.74 | 0.68 (0.39-1.18) | 0.17 | 0.91 (0.70-1.67) | 0.45 |
| RWT | 0.90 (0.70-1.16) | 0.41 | 1.10 (0.64-1.87) | 0.74 | 0.66 (0.38-1.14) | 0.14 | 0.90 (0.70-1.16) | 0.41 |
| SV index to BSA | 0.98 (0.75-1.27) | 0.87 | 1.11 (0.65-1.90) | 0.70 | 0.81 (0.44-1.48) | 0.49 | 0.98 (0.75-1.27) | 0.87 |
| EF (%) | 0.93 (0.72-1.20) | 0.57 | 1.08 (0.63-1.85) | 0.78 | 0.69 (0.39-1.22) | 0.20 | 0.93 (0.72-1.20) | 0.57 |
| LAA 4ch index to BSA | 0.94 (0.72-1.21) | 0.62 | 1.11 (0.65-1.89) | 0.71 | 0.59 (0.32-1.08) | 0.09 | 0.94 (0.72-1.21) | 0.62 |
| RAA 4ch index to BSA | 0.87 (0.67-1.12) | 0.29 | 1.04 (0.61-1.78) | 0.88 | 0.68 (0.38-1.22) | 0.19 | 0.87 (0.67-1.13) | 0.29 |
| E/A Ratio | 0.96 (0.68-1.35) | 0.81 | 1.24 (0.58-2.67) | 0.58 | 0.79 (0.40-1.56) | 0.49 | 0.96 (0.68-1.35) | 0.81 |
| E/e' average | 1.05 (0.79-1.40) | 0.71 | 1.29 (0.71-2.35) | 0.41 | 0.69 (0.35-1.37) | 0.29 | 1.05 (0.79-1.40) | 0.71 |
| MAPSE | 0.93 (0.71-1.22) | 0.61 | 1.10 (0.65-1.89) | 0.72 | 0.71 (0.40-1.28) | 0.26 | 0.93 (0.71-1.22) | 0.61 |
| TAPSE | 0.93 (0.72-1.22) | 0.61 | 1.10 (0.64-1.88) | 0.73 | 0.69 (0.39-1.23) | 0.21 | 0.93 (0.72-1.22) | 0.61 |
| MR change – absolute | 0.92 (0.71-1.18) | 0.51 | 1.11 (0.65-1.89) | 0.71 | 0.61 (0.34-1.11) | 0.10 | 0.92 (0.71-1.18) | 0.51 |
| MR change by >2 grades | 0.92 (0.71-1.18) | 0.51 | 1.05 (0.62-1.80) | 0.85 | 0.66 (0.37-1.15) | 0.14 | 0.92 (0.71-1.18) | 0.51 |
| AR change – absolute | 0.92 (0.71-1.19) | 0.54 | 1.10 (0.65-1.89) | 0.72 | 0.67 (0.38-1.17) | 0.16 | 0.92 (0.71-1.19) | 0.54 |
| AR change by >1 grade | 0.92 (0.72-1.19) | 0.54 | 1.11 (0.65-1.89) | 0.71 | 0.68 (0.39-1.19) | 0.17 | 0.92 (0.72-1.19) | 0.54 |
| AR change by >2 grades | 0.93 (0.72-1.19) | 0.55 | 1.11 (0.65-1.91) | 0.70 | 0.68 (0.39-1.21) | 0.19 | 0.93 (0.72-1.19) | 0.55 |
| TR change – absolute | 0.92 (0.72-1.19) | 0.54 | 1.10 (0.65-1.89) | 0.72 | 0.62 (0.36-1.10) | 0.10 | 0.92 (0.72-1.19) | 0.54 |
| TR change by >1 grade | 0.92 (0.72-1.19) | 0.54 | 1.10 (0.64-1.88) | 0.73 | 0.68 (0.39-1.20) | 0.18 | 0.92 (0.72-1.19) | 0.54 |
| PASP | 1.04 (0.77-1.40) | 0.82 | 0.97 (0.54-1.74) | 0.91 | 0.69 (0.33-1.44) | 0.32 | 1.04 (0.77-1.40) | 0.82 |
| GLS | 0.91 (0.70-1.19) | 0.50 | 1.12 (0.64-1.95) | 0.70 | 0.67 (0.37-1.21) | 0.18 | 0.91 (0.70-1.19) | 0.50 |

Each hazard ratio derived from a multivariable cox proportional hazards regression analysis which adjusts for the change in the relevant echocardiographic variable from baseline to 1 year. Sex coded as male = 1 and female = 0 such that a Hazard Ratio of 1.10 indicates that the hazard of death is 1.10 times greater in males compared to female. AR – aortic regurgitation, BSA – body surface area, E/A – mitral inflow E:A ratio, EF – ejection fraction, GLS – global longitudinal strain, IVSd – interventricular systolic wall thickness in diastole, LAA – left atrial area, MAPSE – mitral annular plane systolic excursion, MR – mitral regurgitation, PASP – pulmonary artery systolic pressure, RAA – right atrial area, RV S’ velocity – right ventricle systolic excursion velocity, RWT – relative wall thickness, SV – stroke volume, TAPSE – tricuspid annular plane systolic excursion.

| Comparison | Regression coefficient | SE | P value |
| --- | --- | --- | --- |
| Sex (males vs female*) | 0.3339 | 0.1099 | 0.002 |
| Genotype (wild-type vs V122I) | 0.0228 | 0.0881 | 0.796 |
| Genotype (T60A vs wild-type*) | 0.2500 | 0.1193 | 0.036 |
| Genotype (T60A vs V122I*) | 0.2728 | 0.1290 | 0.035 |

**Supplementary table A – Multivariable linear regression analysis of interventricular septum in diastole (IVSd) indexed to height**

The regression coefficient represents the estimated difference in means between the two groups. Sex and genotype are coded such that a coefficient >0 indicates that the mean IVSd indexed to height was higher in the second variable compared to the first variable and when adjusted for other variables in the model. *Significant comparator. BSA – body surface area, IVSd – interventricular septum in diastole. SE – standard error

**Supplementary table B – Multivariable linear regression analysis of interventricular septum in diastole (IVSd) indexed to body surface area (BSA)**

| Comparison | Regression coefficient | SE | P value |
| --- | --- | --- | --- |
| Sex (male vs female*) | 0.7712 | 0.1237 | <0.001 |
| Genotype (wild-type vs V122I) | 0.0751 | 0.9917 | 0.449 |
| Genotype (T60A vs V122I) | 0.0130 | 0.1452 | 0.929 |
| Genotype (T60A vs wild-type) | 0.0621 | 0.1346 | 0.644 |

The regression coefficient represents the estimated difference in means between the two groups. Sex and genotype are coded such that a positive coefficient >0 indicates that the mean IVSd indexed to BSA was higher in the second variable compared to the first variable and when adjusted for other variables in the model. *Significant comparator. BSA – body surface area, IVSd – interventricular septum in diastole, SE – standard error

**Supplementary Figure 1 – Proportion of males and females within tertiles of both non-indexed and indexed interventricular septum in diastole (IVSd)**


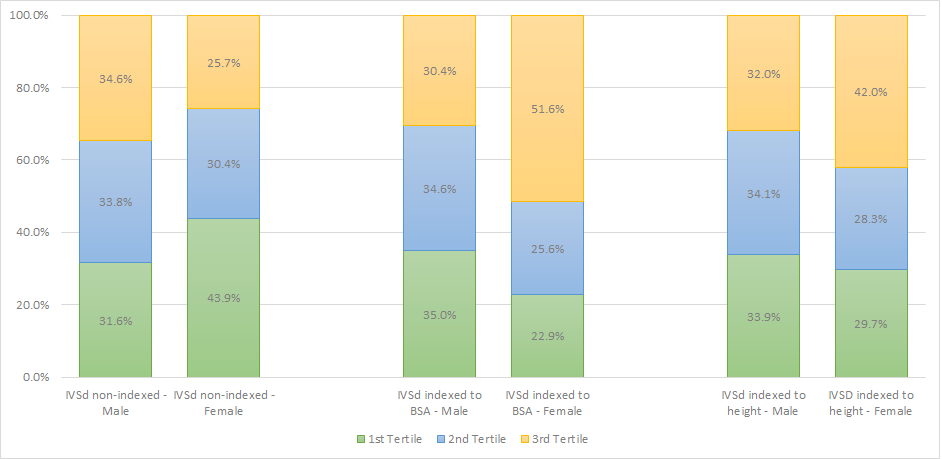

Supplement: Supplementary file 1 — Appendix S1. Supporting Information [file EJHF-24-2355-s001.docx]
